# *Ptip* and the *Trr*-COMPASS-like Complex Regulate Cardiac Progenitor Cell Division in the *Drosophila* Embryonic Heart Tube

**DOI:** 10.3390/ijms26167954

**Published:** 2025-08-18

**Authors:** Adam J. Farmer, Mark H. Inlow, Shaad M. Ahmad, Kristopher R. Schwab

**Affiliations:** 1 Department of Biology, Indiana State University, Terre Haute, IN 47809, USA; farmadam@iu.edu (A.J.F.); shaad.ahmad@indstate.edu (S.M.A.); 2 Rich and Robin Porter Cancer Research Center, Indiana State University, Terre Haute, IN 47809, USA; 3 The Center for Genomic Advocacy, Indiana State University, Terre Haute, IN 47809, USA; mark.inlow@indstate.edu; 4 Department of Mathematical Sciences, Indiana State University, Terre Haute, IN 47809, USA

**Keywords:** *Ptip*, *trr*, *trx*, *Set1*, MLL3/4, KMT2C/D, COMPASS complex, cardiogenesis, heart development and disease, Kabuki Syndrome, *Drosophila*, cardiac progenitor cell division

## Abstract

The Complex Proteins Associated with Set1 (COMPASS)-like complex regulates developmental gene expression via histone 3 lysine 4 (H3K4) methylation and other transcriptional mechanisms. Several members of the lysine methyltransferase 2C and D (KMT2C/D)-COMPASS-like complex are implicated in human congenital heart and vascular defects. The investigation of the orthologous Trithorax-related (Trr)-COMPASS-like complex in *Drosophila melanogaster* (the fruit fly) offers a versatile model to explore gene function in the developing heart. Previous studies have demonstrated the importance of the genes encoding complex members in the later stages of heart development and heart function in both insect and mammalian models. In this study, we investigate the function of *trr* and the complex member *PAX transcription activation domain interacting protein (Ptip)* within the *Drosophila* embryonic dorsal vessel (heart tube). The loss of activity of either gene results in cardiac cell division defects in the Tinman (Tin) and Seven up (Svp) lineages. Furthermore, genetic interaction studies identify a strong synergistic interaction between *Ptip* and *trr* implicating Ptip–Trr–COMPASS-like complex regulation in cardiac progenitor cell division. Interestingly, global H3K4 mono-methylation (H3K4me1) and di-methylation (H3K4me2) levels were not significantly affected in either *Ptip* or *trr* mutants, suggesting that these proteins regulate cardiac target genes at a local scale. In conclusion, these results suggest that the Trr/KMT2C/D–COMPASS-like complex is a key regulator of cardiac progenitor cell division during early embryonic heart development.

## 1. Introduction

Gene expression during heart development is partially controlled by the evolutionarily conserved chromatin-modifying COMPASS-like complexes, which deposit histone 3 lysine 4 methylation modifications at cardiac gene loci [1,2,3,4,5,6]. In addition to heart development, these complexes are important transcriptional regulators that control diverse biological processes, including pluripotency, embryonic development, differentiation, cellular phenotype stability, and oncogenesis [7,8].

Each COMPASS-like complex consists of a SET-domain-containing histone methyltransferase (HMT) of the SET domain containing 1 (Set1) or mixed lineage leukemia (MLL/KMT2) family and several core structural proteins possessing unique H3K4 methylation and transcriptional regulation activities [3,9,10,11]. The SET1-COMPASS-like family, consisting of *Drosophila Set1* and the mammalian orthologous genes *SET1A/B*, maintains global levels of H3K4 di-methylation (H3K4me2) and tri-methylation (H3K4me3) ensuring optimal chromatin accessibility and transcription, while the KMT2–COMPASS-like family imparts various levels of H3K4 methylation to poised and transcriptionally active enhancer, promoter, and gene body regions [12,13,14]. The KMT2 family includes *Drosophila trithorax (trx)*, the ortholog of the mammalian *KMT2A/B (MLL1/2)* genes, and *trithorax-related (trr)*, the ortholog of the mammalian *KMT2C/D (MLL3/4)* genes. The *KMT2A/B* and *trx* gene family members have maintained their evolutionarily conserved role as positive regulators of *Hox* expression in embryonic axial segment determination [15,16,17,18]. Most recently, *trx* has been characterized as a key regulator of cardiac Hox expression and heart patterning within the *Drosophila* embryonic heart tube [19]. In humans, mutations in *KMT2A* and other complex members have been identified in patients with congenital heart defects [20,21,22,23]. The *KMT2C/D* and *trr* gene family regulates diverse biological processes, including embryonic development, cell differentiation, and disease via the activation and maintenance of target gene expression [7,24,25]. Numerous mutations within several KMT2D complex genes are associated with congenital heart defects and other developmental syndromes, which are collectively described as a “congenital regulopathy” due to the likely disruption of developmental gene regulation in many organ systems [26]. For example, haploinsufficiency of *KMT2D* has been identified as a mutation responsible for Kabuki syndrome, a congenital disorder characterized by growth and developmental delays affecting multiple organ systems, including the cardiovascular system [27,28]. In the mouse model, *KMT2D* heterozygosity results in mild anatomical changes to the outflow tract, while inactivation of *KMT2D* induces a wide range of heart defects dependent upon the time of inactivation during heart development [29].

Since a combination of mammalian model and clinical genomic evidence supports a critical role for COMPASS-like function in heart development, the investigation of genetic interactions among components of these complexes will enhance our understanding of the contribution of these genes to congenital heart defects. Each COMPASS-like complex is composed of shared and unique protein subunits necessary for complex stabilization and activity. *Ptip*, a unique protein cofactor of the KMT2C/D complex, encodes a ubiquitously expressed BRCT-domain-containing protein necessary for complex formation, recruitment, and H3K4 methylation [3,9,11]. Ptip and KMT2C/D are actively recruited to promoters and enhancers of transcribed target genes in differentiating and terminally differentiated cells [3,30,31,32,33]. Furthermore, *Ptip* is an important regulator of cardiovascular development. Loss of *Ptip* function results in defects in early heart development and vascularization [3,34,35,36]. In the adult mouse heart, the conditional inactivation of *Ptip* within cardiomyocytes reduces the global levels of H3K4me3 and disrupts the normal cardiac gene expression signature by reducing the expression of several cardiac genes critical for electrolyte, electrochemical homeostasis, and stress response [37,38].

These studies suggest that the Ptip and the KMT2C/D COMPASS-like complex may regulate cardiac gene expression throughout heart development, maturation, and disease; however, further investigation of these genes in a genetically amenable model organism is necessary to characterize these interactions. The *Drosophila melanogaster* embryonic dorsal vessel (heart tube) offers an excellent model to study embryonic heart development due to its precise cellular patterning and quantifiable nature [19,39,40,41,42,43]. Due to an evolutionarily conserved cardiac developmental gene network, the heart tube allows the genetic investigation of candidate gene function in heart development and disease [39,44,45,46,47]. This study investigates the developmental function of *Ptip* in heart development and assesses its role as a core component of the KMT2C/D–COMPASS-like complex. Here, we identify the *Drosophila Ptip* and *trr* as essential regulators of cardiac progenitor cell division in embryonic heart development. Numerous cardiac cell division errors were identified within the embryos lacking *Ptip* or *trr* gene function. Next, we demonstrate that *Ptip* and *trr* genetically interact to synergistically regulate proper cardiac cell division in the embryonic heart, thereby providing evidence to support a role for Ptip-KMT2C/D regulation of cardiac progenitor proliferation and differentiation in mammalian heart development. Although *Ptip* and *trr* have been previously reported to be necessary for global H3K4 methylation, both H3K4me1 and H3K4me2 levels appeared globally normal within mutant heart tubes, suggesting the KMT2D–COMPASS-like complex may act locally at target cardiac genes in embryonic development rather than maintain bulk histone methylation [1,5,6]. Together, these results provide evidence of PTIP-KMT2C/D-COMPASS-like complex action in cardiac progenitor cell division, implicating the possible dysregulation of these genes in congenital heart defect risk in humans.

## 2. Results

### 2.1. Drosophila Ptip Regulates Proper Cardiac Cell Division

The *Drosophila Ptip* gene encodes an essential component of the Trr-COMPASS-like complex that catalyzes H3K4 methylation marks associated with actively transcribed chromatin and is an essential regulator of transcription [3,4]. The *Drosophila trr* gene encodes the HMT of the complex, is responsible for H3K4 methylation, and is an ortholog of the human *KMT2D* gene. *KMT2D* loss-of-function mutations are the genetic cause of Kabuki syndrome, a congenital developmental disorder that affects cardiovascular development [27,28,29,48]. Our previous investigations of the embryonic *Drosophila* heart tube revealed that the COMPASS-like HMTs *trx*, *trr*, and *Set1* independently control cardiac progenitor cell division [19]. Furthermore, both Trr and its specific cofactor Ptip are necessary for cardiac gene expression and histone methylation within both the larval and adult heart [1,5,6]. However, the role of these genes in embryonic heart development had yet to be thoroughly characterized. This study rigorously evaluates both the effects of *Ptip* and *trr* loss-of-function and their genetic interaction within the *Drosophila* embryonic dorsal vessel, thereby characterizing the importance of Trr-COMPASS-like activity in regulating early embryonic heart development.

Since the previously described *Ptip* mutant allele (*Ptip^c04574^*) from the Exelixis collection is no longer available [2], two mutant *Ptip* alleles were identified and obtained for this study. The first was the *Ptip^MI03338^* allele, which contains a MiMIC transposon cassette insertion within exon 4 of *Ptip* predicted to truncate the protein within the glutamine (Q)-rich domain (Supplementary Figure S1) [49]. Furthermore, this *Ptip^MI03338^* insertion is located 5’ of another mutant allele, *Ptip^3804^* [50]. Note that *Ptip^3804^* had previously been reported to be a null mutation because it, too, results in a truncated protein and because two deficiencies that overlap at the *Ptip* gene fail to complement the recessive lethal phenotype of this mutation [50]. Similarly, the *Ptip^MI03338^* allele was also crossed to either of two *Ptip*-overlapping deficiency strains, *Df(3L)ED4515* and *Df(3L)ED4536*, and failed to rescue the pre-pupal lethality phenotype, thereby indicating that the *Ptip^MI03338^* allele is, if not null, a hypomorphic allele. The second *Ptip* loss-of-function allele, *Ptip^c450^* obtained for this study was generated by *CRISPR/Cas9*-mediated deletion of the exon 1 and 2 junction (Supplementary Figure S1) [51]. This mutation was validated by crossing the allele to the *Ptip^MI03338^* MiMIC allele and verifying that the *Ptip^c450^*/*Ptip^MI03338^* genotype also failed to rescue the lethality phenotype. Therefore, the *Ptip^MI03338^* and *Ptip^c450^* alleles were used in this study to characterize the *Ptip* inactivation within the embryonic heart tube and assess potential genetic interactions with the COMPASS-like HMT genes.

**Figure 1:**
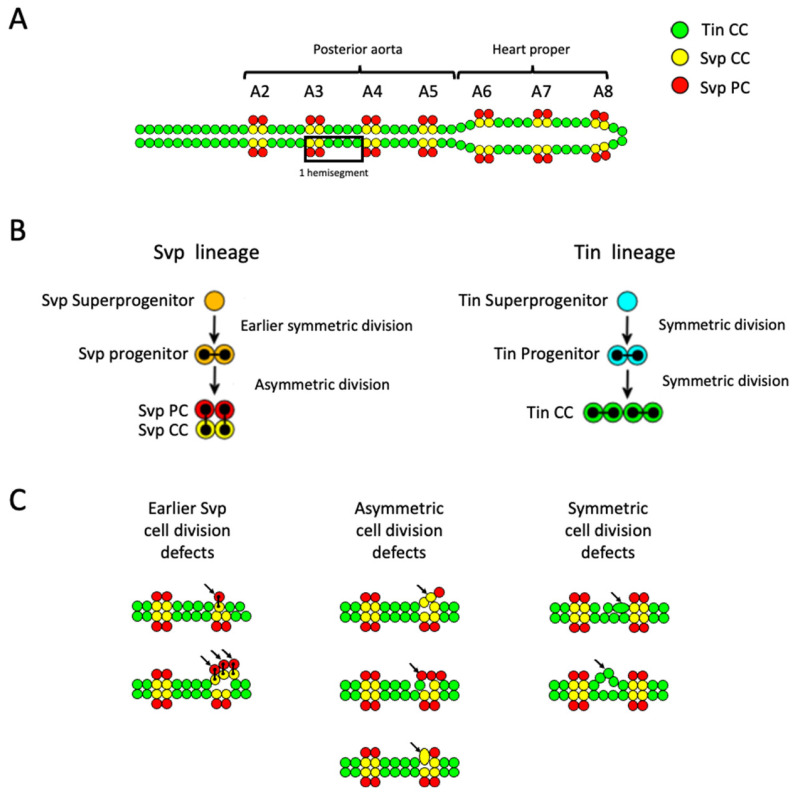
Schematic of the stage 16 wild-type *Drosophila* embryonic heart, cell lineage relationships, and phenotypes due to cardiac cell division defects. (**A**) The posterior aorta and heart-proper regions exhibit the repeated arrangement of two pairs of Svp CCs (yellow) followed by four pairs of Tin CCs (green), which comprise a cardiac hemisegment (box). Svp PCs (red) are located outside the heart tube near their respective Svp CC siblings. (**B**) Lines connect daughter cells arising from the division of each progenitor cell in a wild-type heart. In the Svp lineage (**left**), a Svp superprogenitor cell initially divides symmetrically to produce two sister Svp progenitor cells that undergo an asymmetric cell division each to generate a total of two Svp CCs and two Svp PCs. In contrast, in the Tin cardiac lineage (**right**), the Tin superprogenitor cell undergoes two rounds of symmetric cell division to generate the four Tin CCs. (**C**) Examples of defects in three distinct categories of cardiac cell division that can be evaluated based on the number of Tin CCs, Svp CCs, and Svp PCs in a hemisegment. (**Left**) Defects at the earlier stage of symmetric cell division in the Svp lineage that produces the two Svp progenitors would result in hemisegments with either one or three pairs of Svp CCs and Svp PCs instead of the customary two pairs. (**Middle**) Defects in Svp lineage asymmetric cell division would result in an increase or reduction in the number of Svp CCs accompanied by a corresponding decrease or increase in the number of Svp PCs, respectively, or larger Svp CC nuclei with missing corresponding Svp PCs due to errors in karyokinesis. (**Right**) Defects in Tin lineage symmetric cell division would generate abnormal numbers of Tin CCs.

The embryonic *D. melanogaster* heart offers an excellent model system to investigate cardiac mesoderm induction, cardiac progenitor specification, and cardiac differentiation at the cellular level of resolution [39,40,41,42,43,44,46]. The stage 16 embryonic heart tube comprises symmetrical repeated metameric structures called cardiac “hemisegments”, composed of contractile cardiac cells (CCs) and pericardial cells (PCs), approximately located along the abdominal segments A2–A8 (Figure 1A). The heart tube is composed of myoepithelial CCs that create the lumen of the tube and produce the contractile force necessary to propel hemolymph through the vessel. PCs are nephrocytic cells that surround the CCs of the heart tube and filter hemolymph. Each hemisegment is symmetrically aligned with its contralateral partner across the midline and contains two *seven up*-expressing cardiac cells (Svp CCs) followed by four *tinman*-expressing cardiac cells (Tin CCs) [52,53,54,55,56]. The cardiac progenitor cells that give rise to the Svp and Tin CC lineages undergo unique patterns of cell divisions. The Tin superprogenitor cell of a hemisegment undergoes two rounds of symmetric cell division to generate the four Tin CCs (Figure 1B, right), while the Svp superprogenitor cell initially divides symmetrically to produce two sister Svp progenitor cells that then undergo an asymmetric cell division each to generate a total of two Svp CCs and two Svp PCs, which remain in close proximity to one another (Figure 1B, left). The repeated and stereotyped configuration of Svp and Tin CCs in the hemisegments of the heart tube and the characteristic patterns resulting from each type of cell division defect enable the identification and quantification of cardiac cell division errors as Svp earlier-superprogenitor, Svp asymmetric, or Tin symmetric cell defects.

For example, if the earlier (initial) symmetric cell division of the Svp superprogenitor cell in a hemisegment is defective, then it may result in either one or three Svp progenitor cells instead of the expected two, which in turn will produce either one pair or three pairs of Svp CCs and Svp PCs via asymmetric cell division. Thus, the presence of just one Svp CC and one Svp PC in a hemisegment, or three Svp CCs coupled with three Svp PCs, will be interpreted as an earlier, symmetric cell division defect in the Svp lineage (Figure 1C, left).

In contrast, if the later asymmetric cell division of one of the two Svp progenitor cells goes awry, then it may produce two Svp CCs or two Svp PCs in place of one Svp CC and one Svp PC. Therefore, the detection of one Svp CC and three Svp PCs, or one Svp PC and three Svp CCS, in place of the expected two Svp CCs and two Svp PCs per hemisegment, would indicate a defect in asymmetric cell division along the Svp lineage (Figure 1C, middle). However, asymmetric cell division defects along the Svp lineage may also include karyokinesis defects where a nucleus is arrested in the process of dividing and appears as one larger Svp CC nucleus instead of one Svp CC nucleus and one Svp PC nucleus [40]. Such defects can be identified as hemisegments with two Svp CCs and one Svp PC (Figure 1C, middle).

Finally, defects in symmetric cell division along the Tin lineage of either the Tin superprogenitors or the Tin progenitor cells can be distinguished as any variation in the number of Tin CCs from the expected four (Figure 1C, right).

We used these aforementioned criteria for categorizing the three classes of cell division defects in this research project.

**Figure 2:**
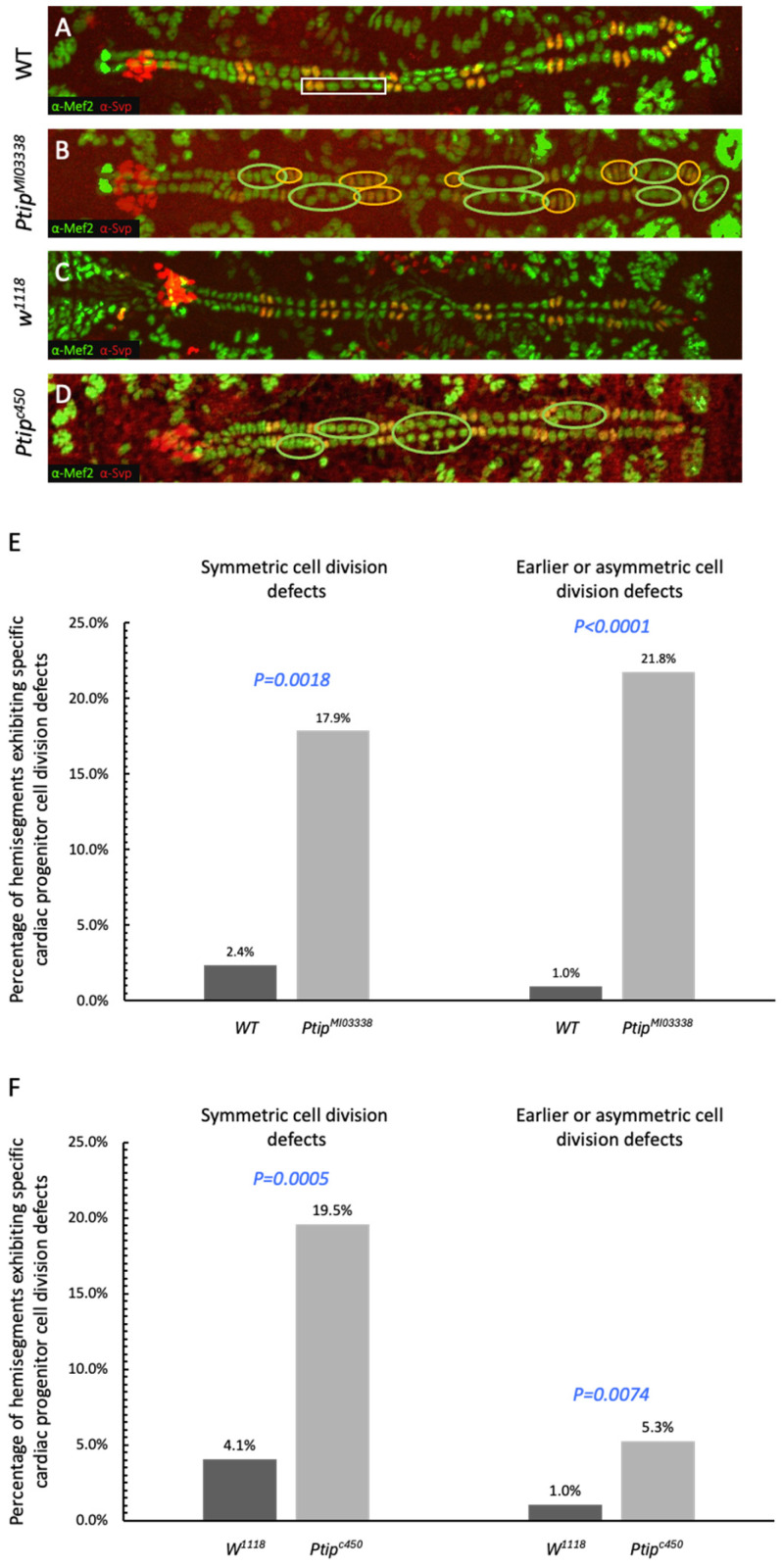
The cardiac cell division defects identified within the *Ptip^MI03338^* and *Ptip^c450^* strains. (**A**) A wild-type embryonic heart immunostained using α-Svp (red) and α-Mef2 (green) antibodies, identifying the characteristic two Svp CCs (yellow) followed by the four Tin CCs (green), respectively, per hemisegment. The white rectangle identifies a typical hemisegment. Note that the Svp PCs are not visible in this assay. (**B**) The heart from an embryo homozygous for the *Ptip^MI03338^* allele exhibits Tin lineage symmetric cell division defects (identified by the green circles) and Svp lineage earlier cardiac progenitor or asymmetric cell division defects (identified by the yellow circles). (**C**) A heart from an otherwise wild-type embryo with the *w^1118^* background. (**D**) The heart tube from an embryo homozygous for the *Ptip^c450^* allele also displays cell division defects. (**E**,**F**) Percentage of hemisegments exhibiting each type of cardiac cell division defect. The statistical significance for each type of cardiac cell division defect in the *Ptip* mutant embryos is compared to that in the appropriate wild-type or control embryos, as noted above the bar graph. (**E**) The *Ptip^MI03338^* mutant heart tube displays significantly increased numbers of each cardiac cell division defect compared to those in the appropriate wild-type control embryos. A total of 210 hemisegments from the hearts of 15 wild-type embryos and 308 hemisegments from 22 embryos for *Ptip^MI03338^* were analyzed. (**F**) Similarly to the *Ptip^MI03338^* results, the *Ptip^c450^* mutant heart exhibits significantly increased percentages of hemisegments with either cell division defect. A total of 266 hemisegments from 19 embryos were analyzed for *Ptip^c450^*, and 196 hemisegments were analyzed from 14 embryos for *w^1118^*.

To investigate the effects of *Ptip* loss of function within the *Drosophila* heart tube, stage 16 homozygous *Ptip^MI03338^* and *Ptip^c450^* mutant embryos were immunostained with anti-Myocyte enhancer factor 2 (Mef2) and anti-Seven up (Svp) antibodies, which label all CCs and specifically Svp CCs, respectively (Figure 2). Tin CCs are identified as Mef2-positive Svp-negative cells in the heart, while Svp CCs are identified as being both Mef2-positive and Svp-positive. It is important to note that the sensitivity of the anti-Svp antibody is insufficient to detect the low Svp protein levels within the Svp PCs. Therefore, Svp PCs are not detectable in this figure. To assess cardiac progenitor cell division defects, the A2-A8 hemisegments were examined and scored for an increase or decrease in the number of both Tin CCs, which reflect errors in symmetrical cell divisions, and Svp CCs, which reflect errors in the earlier symmetric and/or later asymmetric cell division compared to the appropriate strain-matched wildtype embryo. The investigation of both *Ptip^MI03338^* and *Ptip^c450^* homozygous embryos identified significant increases in cell division defects over the controls (Supplementary Table S1). In the *Oregon R* wild-type strain, only 2.4% of the hemisegments exhibited Tin-lineage symmetric cell division defects, while 1% of the hemisegments displayed Svp-lineage earlier-progenitor or asymmetric cell division defects. In contrast, our analysis of the *Ptip^MI03338^* mutant embryos revealed that 17.9% and 21.8% of the hemisegments exhibited Tin-lineage symmetric (*p* = 0.0018) and Svp-lineage earlier progenitor or asymmetric cell division (*p* < 0.0001) defects, respectively (Figure 2A, B, and E).

The *Ptip^c450^* allele was derived from the *w^1118^* background, a strain commonly used for genetic engineering [51]. Our analysis also detected statistically significant increases in cell division defects for the *Ptip^c450^* strain in both the Tin- (*p* = 0.0005) and Svp-lineages (*p* = 0.0074) compared to otherwise wild-type *w^1118^* embryos (Figure 2C,D,F). Overall, these findings indicate that *Ptip* function is necessary for normal cardiac progenitor cell divisions of the Tin and Svp cardiac lineages and implicate a possible role for the Ptip-trr COMPASS-like complex in the regulation of cardiac progenitor cell division.

### 2.2. Ptip and Trr Regulate Tin-Lineage Symmetric and Svp-Lineage Earlier Cardiac Progenitor Cell Divisions

**Figure 3:**
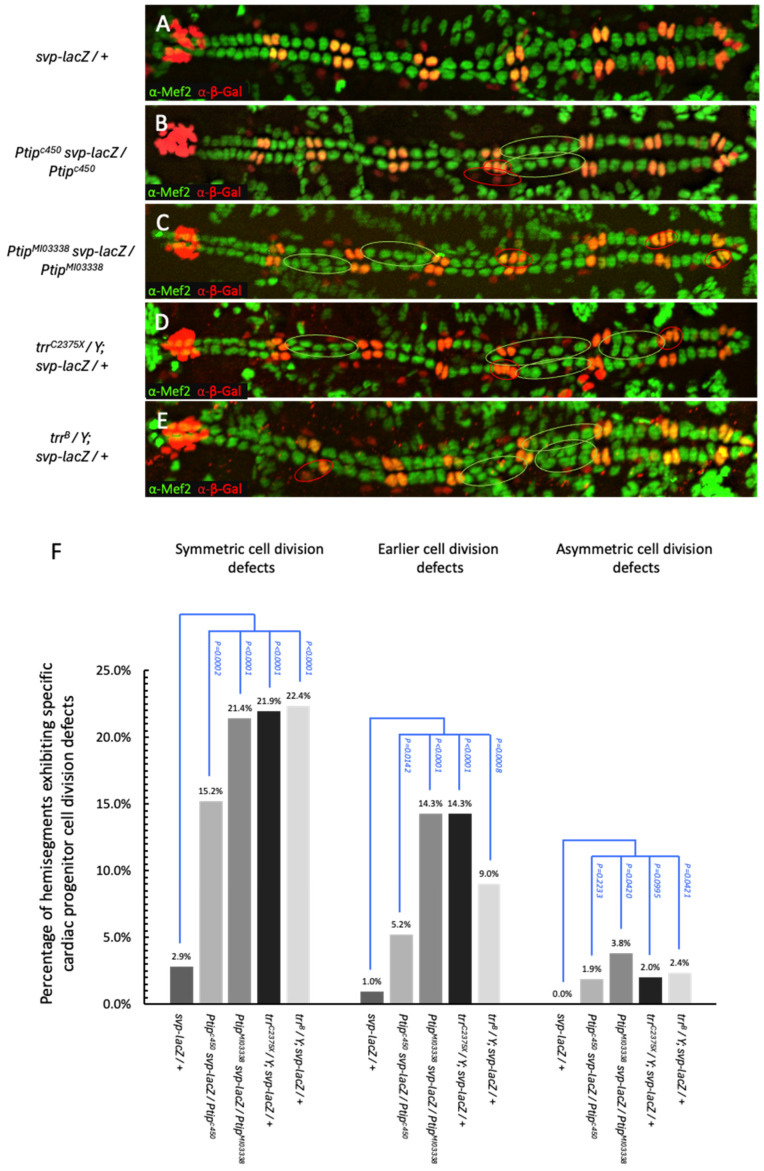
Cardiac cell division defects within both *Ptip* and *trr* mutant strains and controls using *svp-lacZ* enhancer trap allele to visualize the Tin CCs, Svp CCs, and Svp PCs. (**A**) A dorsal vessel from a control embryo (*svp-lacZ/+*) bearing one copy of the *svp-lacZ* enhancer trap allele immunostained using anti-β-galactosidase (red) and anti-Mef2 (green) antibodies identifies the characteristic two Svp CCs (yellow) followed by the four Tin CCs (green), respectively, per hemisegment. The two Svp PCs (red) are visible outside of the epithelial heart tube near their corresponding Svp CCs. Note that since these images were derived by flattening z-stacks, some Svp PCs may be faint or not visible due to their position below the plane of the heart tube. (**B**–**E**) Images from homozygous *Ptip* or *trr* mutants in a *svp-lacZ* background. Examples of cardiac cell division defects in the Tin CC lineage (green circles) and Svp CC/PC lineage (red circles) are shown for the following genotypes: (**B**) *Ptip^c450^ svp-lacZ/Ptip^c450^*, (**C**) *Ptip^MI03338^ svp-lacZ/Ptip^MI03338^*, (**D**) *trr^C2375X^/Y; svp-lacZ/+*, and (**E**) *trr^B^/Y; svp-lacZ/+*. As in (**A**), some Svp PCs may be faint or not visible due to their position below the plane of the heart tube. (**F**) Percentage of hemisegments exhibiting each type of cardiac cell division errors: Tin-lineage symmetric cell division defects, defects in Svp-lineage of early and symmetric cell division of superprogenitor cells, and Svp-lineage asymmetric cell division defects. The statistical significance for the frequency of each type of cardiac cell division defect of the mutant embryos compared to the *svp-lacZ/+* control embryo is presented. A total of 196 hemisegments from 14 embryos each were analyzed for the *svp-lacZ/+* and *trr^C2375X^/Y*; *svp-lacZ/+* groups; a total of 210 hemisegments from 15 embryos were analyzed for the remaining genotypes.

The Svp CCs and PCs of the embryonic heart tube are generated by a series of successive cell divisions. As described previously, a single Svp early cardiac superprogenitor cell divides symmetrically to produce two Svp progenitor cells. Both Svp progenitor cells subsequently undergo asymmetric cell division to generate the two Svp CCs within the heart tube and two outer nephrocytic Svp PCs (Figure 1B). The perdurance of the β-galactosidase from the *svp-lacZ* enhancer trap allele enables the visualization of both Svp CCs and Svp PCs and can be utilized to classify the Svp-lineage cell division errors as defects in either the division of the earlier Svp superprogenitor cell or in the later asymmetric cell division of the twin Svp progenitors [55,56,57] (Figure 1C and Figure 3). Tin CCs are identified as Mef2-positive β-galactosidase-negative cells in the heart, Svp CCs as Mef2-positive β-galactosidase-positive cells, and Svp PCs as Mef2-negative β-galactosidase-positive cells.

We quantified both categories of Svp-lineage cell division defects, as well as the Tin-linage cell division defects, occurring within the A2-A8 hemisegments of stage 16 homozygous *Ptip* mutant embryos (Supplementary Table S2). To evaluate the *Ptip* mutants in this manner, recombinant *Ptip svp-lacZ/Ptip* embryos and appropriate controls consisting of *svp-lacZ/+* embryos were generated and immunostained with anti-Mef2 and anti-β-Galactosidase antibodies to visualize the Mef2 protein and *svp-lacZ* reporter activity, respectively.

Our results in the Tin lineage that led to the production of Tin CCs were similar to the data reported in Figure 2. Symmetric cell division defects in the Tin lineage were significantly higher in the *Ptip^c450^* (15.2%, *p* = 0.0002) and *Ptip^MI03338^* (21.4%, *p* < 0.0001) recombinant embryos compared to those (2.9%) in the *svp-lacZ*-bearing otherwise wild-type controls (Figure 3A–C,F).

Among the two distinct types of cell division defects in the Svp lineage, errors in the earlier symmetric cell divisions of the Svp superprogenitors were also significantly increased in *Ptip^c450^* (5.2%, *p* = 0.0142) and *Ptip^MI03338^* (14.3%, *p* < 0.0001) mutants over those (1.0%) in the controls (Figure 3A–C,F).

However, the prevalence of the other category of Svp-lineage cell division defects, i.e., errors in asymmetric cell division of the Svp progenitors, was inconsistently significant between the *Ptip^c450^* (1.9%, *p* = 0.2233) and *Ptip^MI03338^* (3.8%, *p* = 0.0420) embryos when compared to controls (0.0%) (Figure 3A–C,F). In summary, both the *Ptip^MI03338^* and *Ptip^c450^* mutant embryos exhibit a significant increase in cell division defects within the Tin lineage and earlier superprogenitor cell division defects in the Svp lineage, indicating that *Ptip* is necessary for proper symmetric cardiac cell division in the dorsal vessel.

Given the molecular and biochemical evidence of the Ptip-dependent recruitment of the trr–COMPASS-like complex to gene regulatory sites and loci undergoing active transcription and enrichment of H3K4 methylation, *trr* is likely to be a synergistic interaction partner with *Ptip* during heart development, particularly in mediating proper cardiac cell divisions. If this hypothesis is true, then loss of function of *trr* should exhibit phenotypes similar to those observed in the *Ptip* mutants. To test this hypothesis, two hypomorphic mutant alleles of *trr* were evaluated for embryonic heart defects at stage 16 using the *svp-lacZ* reporter system. The *trr^C2375X^* allele is predicted to truncate the protein within the C-terminal SET domain, while the *trr^B^* allele is predicted to terminate earlier in translation at amino acid 512, thereby removing most of the protein sequence (Supplementary Figure S1) [58,59,60]. *trr^C2375X^/Y*; *svp-lacZ/+* and *trr^B^/Y*; *svp-lacZ/+* hemizygous mutant embryos were immunostained to visualize the Tin- and Svp-lineage cardiac cell division defects within the A2–A8 hemisegments in a similar manner to that in the *Ptip* mutants. The comparison of the *trr^C2375X^* and *trr^B^* hemizygous embryos reveal a similar percentage of hemisegment defects for each allele in each of the three categories of cardiac cell divisions: Tin-lineage symmetric cell division defects (21.9%, *p* < 0.0001 and 22.4%, *p* < 0.0001, respectively), Svp-lineage earlier symmetric superprogenitor cell division defects (14.3%, *p* < 0.0001 and 9.0%, *p* = 0.0008, respectively), and Svp-lineage asymmetric cell division defects (2.0%, *p* = 0.0995 and 2.4%, *p* = 0.0421, respectively) compared to their appropriate controls (Figure 3A,D,E,F; Supplementary Table S2).

Overall, this analysis indicates that the hemizygous *trr* mutant heart tube phenocopies the homozygous *Ptip* mutant phenotypes, consistent with our hypothesis that *Ptip* and *trr* may be mediating the same cardiogenic pathway, specifically subsets of cardiac cell division. Indeed, both mutant alleles of *Ptip* and both mutant alleles of *trr* exhibit significantly more defects than control embryos for Svp-lineage earlier superprogenitor and Tin-lineage progenitor cell divisions, demonstrating a requirement for both these genes in symmetric cardiac cell divisions. In contrast, in the case of either *Ptip* or *trr* mutant alleles, the change in defects in Svp-lineage asymmetric progenitor cell divisions was barely significant for one allele, and not significant in the case of the other allele. Collectively, this latter data suggests that both *Ptip* and *trr* might not be essential for mediating asymmetric cardiac cell divisions. This remarkable similarity between the cardiac cell division subtypes mediated by *Ptip* and *trr* lends further support to our hypothesis that these two genes function through the same pathway to regulate aspects of cardiac cell division.

### 2.3. Genetic Interaction Studies Demonstrate a Synergistic Interaction Between PTIP and Trr in Cardiac Cell Division Regulation

Based upon this remarkable similarity between the *Ptip* and *trr* mutant cardiac cell division phenotypes that we report and the Ptip–trr–COMPASS-like complex interactions previously identified in both fly and mammalian studies, we hypothesized that these genes synergistically regulate cardiac cell division within the embryonic dorsal vessel. The Ptip-Trr–COMPASS-like complex has been demonstrated to be essential for global, cis-regulatory, and gene locus-specific H3K4 methylation associated with open and actively transcribed chromatin. The loss of either gene or protein diminishes the regulatory activity of the complex. Thus, if the Ptip-Trr COMPASS-like complex also mediated cardiac cell divisions, then the products of *Ptip* and *trr* would be expected to interact to regulate the cell cycle, explaining why the reduction in normal gene dosage level of both genes interfered with cardiac cell division in an identical manner.

**Figure 4:**
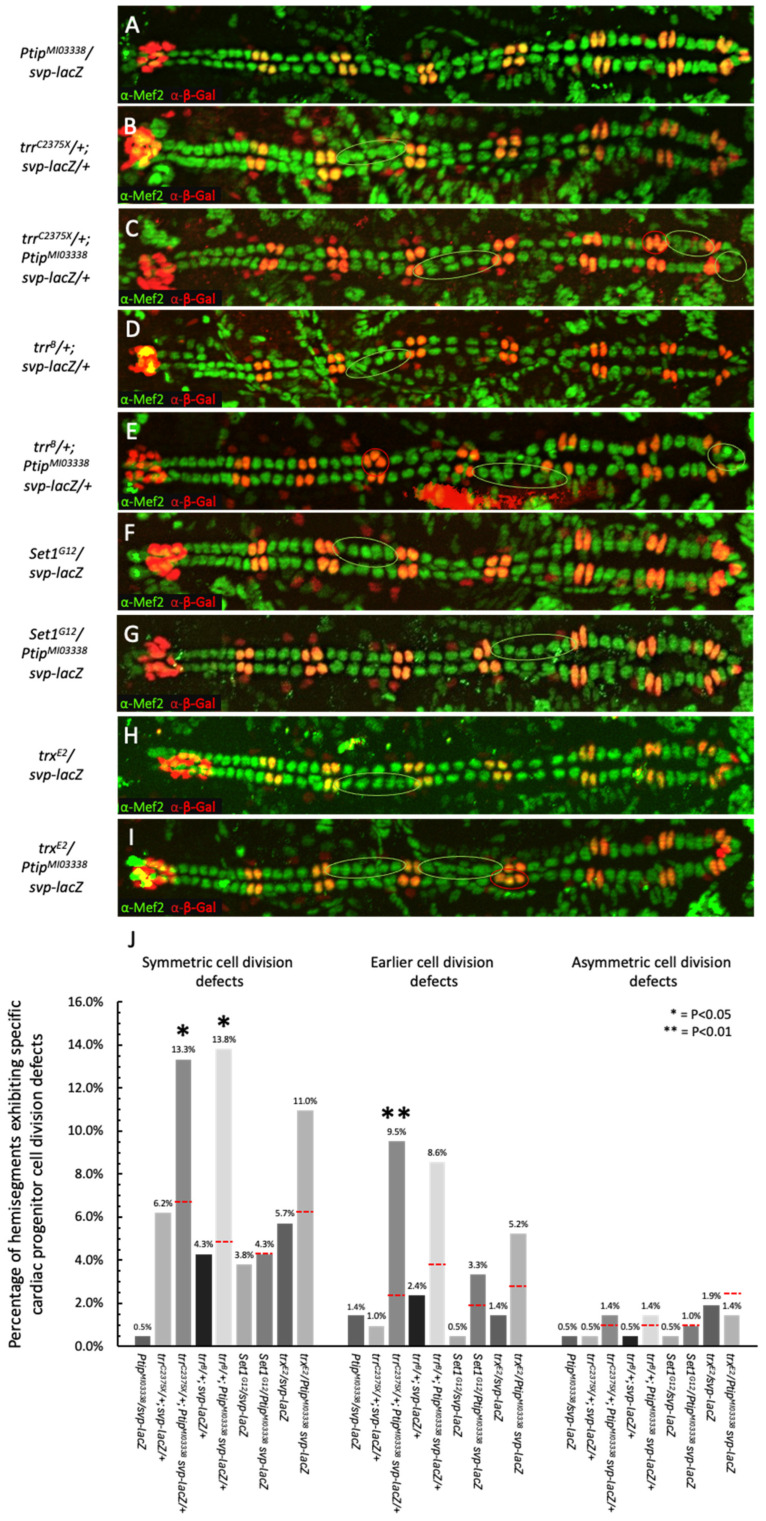
*Ptip* exhibits synergistic genetic interactions with *trr* but not with *Set1* or *trx* in regulating cardiac cell divisions. (**A**–**E**) Representative images of heart tubes from embryos singly or doubly heterozygous for mutations of *Ptip* and *trr* immunostained using anti-β-galactosidase (red) and anti-Mef2 (green) antibodies to identify the Svp CCs (yellow), Tin CCs (green), and Svp PCs (red). Note the increase in apparent cell division defects in the double heterozygotes as compared to the single heterozygotes of *Ptip* or *trr* alleles. (**F**–**I**) Representative heart tubes from embryos singly heterozygous for mutations in *Set1* and *trx*, and doubly heterozygous for mutations in both *Set1* and *Ptip* and both *trx* and *Ptip*. Examples of cardiac cell division defects in the Tin CC lineage (green circles) and Svp CC/PC lineage (red circles) are shown for the following genotypes: (**A**) *Ptip^MI03338^/svp-lacZ*, (**B**) *trr^C2375X^/+; svp-lacZ/+*, (**C**) *trr^C2375X^/+*; *Ptip^MI03338^ svp-lacZ/+*, (**D**) *trr^B^/+*; *svp-lacZ/+*, (**E**) *trr^B^/+*; *Ptip^MI03338^ svp-lacZ/+*, (**F**) *Set1^G12^/svp-lacZ*, and (**G**) *Set1^G12^/Ptip^MI03338^ svp-lacZ*, (**H**) *trx^12^/svp-lacZ*; and (**I**) *trx^1E2^/Ptip^MI03338^ svp-lacZ.* (**J**) Quantitation of cardiac cell division defects in the single and double heterozygotes. The red dashed lines in the bars for the double heterozygotes indicate the expected percentage of specific cell division errors if they were purely the additive sum of the percentages in the relevant single heterozygotes. Note that our results indicate that *Ptip* and *trr* genetically interact in a synergistic manner to facilitate Tin-lineage symmetric cell divisions for both *trr* alleles used in this study, i.e., the percentage of cell division defects in each of the double heterozygotes is significantly greater than the additive effect (red dashed line) of the cell division errors of the individual single heterozygotes. Synergistic genetic interactions are also detected between *Ptip* and *trr* in regulating earlier symmetrical Svp-lineage superprogenitor cell divisions for one of the two *trr* alleles. In contrast, no synergistic genetic interactions are detected between *Ptip* and *Set1* or *Ptip* and *trx* for any of the three categories of cardiac cell division. A total of 210 hemisegments from 15 embryos were analyzed for each genotype.

To investigate potential genetic interactions between *Ptip* and *trr* within the heart tube, female embryos doubly heterozygous for mutations in both genes and possessing the *svp-lacZ* enhancer trap allele were evaluated for defects in each of the three categories of cardiac cell division—symmetric cell division in the Tin-lineage, earlier superprogenitor cell division in the Svp-lineage, and asymmetric progenitor cell division in the Svp-lineage—and compared to single heterozygotes for these mutations that also carried *svp-lacZ* (Figure 4A–E,J; Supplementary Table S3). If Ptip and Trr actually function as a complex to mediate a specific subset of cardiac cell divisions, then we may expect to detect synergistic genetic interaction between the *Ptip* and *trr* genes. In our assay, such synergistic genetic interactions would be detected as the percentage of specific cell division defects within the double heterozygotes being significantly greater than the additive sum of the frequencies of cell division errors in both of the single heterozygous conditions. However, if the two genes act independently of the Ptip-Trr COMPASS-like complex or indirectly regulate cardiac cell division, then the resulting defects within the double heterozygous condition would be merely additive or less than the sum of the defects found in the single heterozygous conditions.

Double heterozygotes comprise the *Ptip^MI03338^* mutant allele and either of the two *trr* mutant alleles exhibited Tin-lineage symmetric cell division defects that were significantly more severe than the additive sum of the defects of *Ptip* mutant single heterozygotes and either of the two *trr* mutant single heterozygotes (*p* = 0.012 when *trr^C2375X^/+*; *Ptip^MI03338^*, *svp-lacZ/+* was compared with *trr^C2375X^/+*; *svp-lacZ/+* and *Ptip^MI03338^/svp-lacZ,* and *p* = 0.010 when *trr^B^/+; Ptip^MI03338^*, *svp-lacZ/+* was compared with *trr^B^/+*; *svp-lacZ/+* and *Ptip^MI03338^/svp-lacZ*) (Figure 4A–E,J). The synergistic genetic interactions observed in these results indicate that Ptip and Trr do indeed function as a complex to mediate symmetric cardiac cell divisions.

A similar synergistic genetic interaction between *Ptip* and *trr* was detected when the mutant alleles *Ptip^MI03338^* and *trr^C2375X^* were used to assess their role in the earlier Svp-lineage superprogenitor cell divisions (*p* = 0.007 when *trr^C2375X^/+; Ptip^MI03338^, svp-lacZ/+,* was compared with *trr^C2375X^/+*; *svp-lacZ/+* and *Ptip^MI03338^/svp-lacZ*) (Figure 4A–C,J). When the mutant alleles *Ptip^MI03338^* and *trr^B^* were used for the same genetic interaction assay, the frequency of earlier Svp-lineage cell division defects for the double heterozygotes (8.6%) was still considerably larger than the additive sum of the frequency of the defects for the single heterozygotes (1.4% for *Ptip^MI03338^/svp-lacZ* and 2.4% for *trr^B^/+; svp-lacZ/+*), but the significance of this genetic interaction being synergistic was borderline (*p* = 0.069) (Figure 4A,D,E,J). Collectively, however, the significant synergistic genetic interaction detected between these two genes using the *trr^C2375X^* mutant allele, and the inconclusive results obtained with the other *trr^B^* mutant allele, clearly suggest that Ptip and Trr likely also function as a complex to mediate earlier Svp-lineage superprogenitor cell divisions.

In contrast, no synergistic genetic interactions were detected at all between the *Ptip* mutant allele and either of the two *trr* mutant alleles for Svp-lineage progenitor asymmetric cell divisions. The frequencies of asymmetric cardiac cell division defects for the double heterozygotes were not significantly larger than the additive sum of those for the individual single heterozygotes (*p* = 0.637 when *trr^C2375X^/+*; *Ptip^MI03338^*, *svp-lacZ/+*, was compared with *trr^C2375X^/+*; *svp-lacZ/+* and *Ptip^MI03338^/svp-lacZ* and *p* = 0.636 when *trr^B^/+*; *Ptip^MI03338^*, *svp-lacZ/+* was compared with *trr^B^/+*; *svp-lacZ/+* and *Ptip^MI03338^/svp-lacZ*) (Figure 4A–E,J), consistent with the idea that the Ptip-Trr COMPASS-like complex might not be critical for regulating asymmetric cardiac progenitor cell divisions.

Collectively, the results of our genetic interaction assays clearly demonstrate a role for the Ptip-Trr–COMPASS-like complex in mediating Tin-lineage cardiac progenitor symmetric cell divisions, argue strongly that this same complex also plays a critical role in regulating the earlier Svp-lineage superprogenitor cell divisions, and are consistent with the complex not being essential for Svp-lineage asymmetric cardiac progenitor cell divisions.

### 2.4. No Synergistic Genetic Interactions Are Detected Between Ptip and Set1 or Between Ptip and Trx in Cardiac Cell Division Regulation

Our previous investigation of the embryonic *Drosophila* heart tube had revealed that all three of the COMPASS-like HMT-encoding genes *trr*, *Set1*, and *trx* regulate cardiac cell divisions [19]. In this study, we have shown that *Ptip* also mediates cardiac cell division. However, the HMTs encoded by *trr*, *Set1*, and *trx* are components of distinct COMPASS-like complexes, while Ptip is known to date to be a member of only the complex associated with Trr. These observations raise the question of whether cardiac cell division is regulated by *Ptip* and *Set1* or *Ptip* and *trx* working in concert through the same genetic/molecular pathways in a manner similar to *Ptip* and *trr* or is completely independent of one another.

In order to determine which of these possibilities were correct, we quantitated and compared the cardiac cell division defect phenotypes of single heterozygotes of a *Ptip* mutation, single heterozygotes of the *Set1* mutation (Supplementary Figure S1), and double heterozygotes comprising both *Ptip* and *Set1* mutations in genetic interaction assays. Similar genetic interaction assays were also performed with single and double heterozygotes of mutations for *Ptip* and *trx* (Supplementary Figure S1). If *Ptip* functions in unison with *Set1* or *trx* to mediate cardiac cell divisions then we may expect to see synergistic genetic interactions between *Ptip* and the relevant HMT-encoding gene. If, on the other hand, Ptip operates completely independently of these two HTMs, then no such synergism would be detected.

For each of the three categories of cardiac cell divisions, we did not detect any synergistic, i.e., more than merely additive, genetic interactions between *Ptip* and *Set1* (*p* = 0.979 for Tin-lineage symmetric cell divisions, *p* = 0.334 for Svp-lineage earlier cell divisions, and *p* = 0.999 for Svp-lineage asymmetric cell divisions) or between *Ptip* and *trx* (*p* = 0.171 for Tin-lineage symmetric cell divisions, *p* = 0.281 for Svp-lineage earlier cell divisions, and *p* = 0.431 for Svp-lineage asymmetric cell divisions) (Figure 4A,F–I,J; Supplementary Table S3). These results are consistent with *Ptip* and *trr* controlling cardiac cell division via processes that are independent of the other two COMPASS-like HTMs.

### 2.5. Global Cardiac Mono- and Di-H3K4 Methylation Is Not Affected by Loss of Ptip or Trr Activity

**Figure 5:**
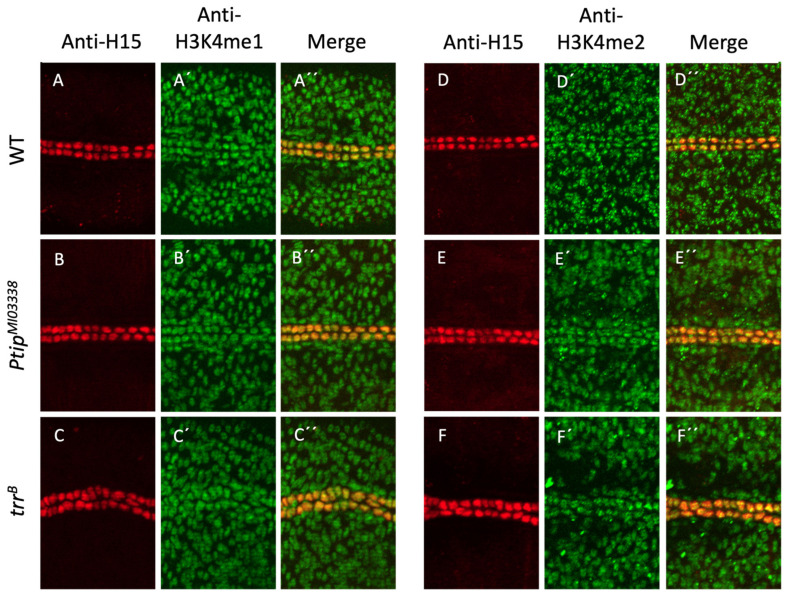
Mono- and di-H3K4 methylation levels are maintained in the *Ptip* and *trr* mutants. Images of wild-type (**A**–**A’’**,**D**–**D’’**), *Ptip* homozygous mutant *(***B**–**B’’**,**E**–**E’’)** and *trr* (**C**–**C’’**,**F**–**F’’**) homozygous mutant embryos immunostained using anti-H15 (red) to identify the CCs of the heart tube (**A**,**B**,**C**,**D**,**E**,**F**) and anti-H3K4me1 (**A’**,**B’**,**C’**) or anti-H3K4me2 (**D’**,**E’**,**F’**) (green) antibodies. (**A’’**,**B’’**,**C’’**,**D’’**,**E’’**,**F’’**) Merged images identifies co-localization of H15 and H3K4 methylation in CCs (yellow) of wildtype (*n* = 3), *Ptip* mutant (*n* = 3), and *trr* mutant (*n* = 3) embryos indicating that global levels of H3K4 methylation are not significantly affected.

Both Ptip and Trr are necessary for H3K4me1 and H3K4me2 methylation via the Trr-COMPASS-like complex acting globally within the chromatin of the nucleus and locally at actively transcribed gene loci [9,10]. To evaluate the roles of both of these genes in global H3K4 methylation, wildtype and relevant mutant embryos were stained for anti-H15 (Neuromancer1, Nmr1) that labeled all CCs and anti-H3K4me1 or anti-H3K4me2 antibodies. Interestingly, both H3K4me1 and H3K4me2 remain globally unaffected in both the whole embryo and heart tube of the *Ptip* and *trr* mutants, suggesting that H3K4 HMTs other than the Trr–COMPASS-like complex maintain global H3K4 levels during embryonic heart development (Figure 5). However, we cannot preclude important chromatin regulatory functions of Ptip and trr at specific target gene loci that may not have been detectable at this level of resolution.

## 3. Discussion

In this study, we describe the cardiogenic functions of *Ptip* and *trr* in *Drosophila*, genes which encode essential components of the Trr-COMPASS-like complex. Early cardiac mesoderm specification appears to be unaffected by the loss of either *Ptip* or *trr* since the overall organization of the cardiac hemisegments remains intact within the mutant embryos. Furthermore, unlike what we had previously observed when another COMPASS-like HMT-encoding gene, *trx*, had been disrupted [19], the microanatomy of the heart tube in these *Ptip* and *trr* mutants appears normal suggesting that *Hox* activity and the anterior–posterior patterning of the heart is also not significantly affected. However, it should be noted that several of the mutant strains utilized in this study may retain partial gene activity based on their predicted effects on gene structure (Supplementary Figure S1). Additionally, we cannot completely eliminate the possibility of a significant *Ptip* or *trr* maternal effect rescuing the loss of zygotic expression within the dorsal vessel during embryogenesis. Unfortunately, *Ptip* mutant embryos derived from germline mutant clones exhibit severe segmentation defects thereby disrupting normal dorsal vessel development, while *trr* mutant embryos derived from germline mutant clones fail to initiate mitotic division after fertilization [2,61]. These effects prevent the conclusive evaluation of the maternal and zygotic functions of *Ptip* and *trr* during mesodermal specification, cardiac mesoderm migration, and cardiac cell differentiation.

Additionally, our ability to evaluate zygotic *Ptip* and *trr* gene functions in heart growth and remodeling during larval heart development is thwarted by the post-embryonic, pre-pupal lethality exhibited within the homozygous mutant individuals.

However, the use of a *svp-lacZ* enhancer trap allele in our studies allowed the precise quantitation of defects in the three types of cell division that comprise the A2–A8 hemisegments of the *Drosophila* embryonic heart: Tin-lineage symmetric cardiac progenitor cell divisions, earlier Svp-lineage superprogenitor cell divisions, and subsequent Svp-lineage asymmetric cardiac progenitor cell divisions. Comparison of *Ptip* mutant hearts and *trr* mutant hearts with those of controls in our studies exhibited significantly abnormal numbers of specific cell division defects which indicated that both of these genes were essential for Tin-lineage symmetric cardiac progenitor cell divisions and earlier Svp-lineage superprogenitor cell divisions. Given that Svp-lineage superprogenitor cell divisions in the developing heart are also considered to be symmetric [52,53,54,55,56], the results of our investigation clearly implicate both *Ptip* and *trr* in symmetric cell division during cardiogenesis.

In contrast, our results from the quantitation of cardiac cell division defects suggest that *Ptip* and *trr* might not make any meaningful contributions to asymmetric Svp-lineage progenitor cell divisions. While one mutant allele for *Ptip* exhibited no significant increase in Svp-lineage asymmetric cell division defects over the control, the other *Ptip* mutant displayed merely borderline significance. Similarly, one of the *trr* mutant alleles showed no significant change in the frequency of Svp-lineage asymmetric cell division defects, while the other was barely significant. The asymmetric cell division of the Svp-lineage cardiac progenitors to produce both Svp CCs and Svp PCs is achieved via the suppression of Notch signaling through Numb accumulation within the Svp PCs [52,53,54,55,56]. Our results thus suggest that the influence of *Ptip* and *trr* in this process is either nonexistent or negligible.

Ptip and Trr are both essential components of just one of three COMPASS-like complexes present in *Drosophila*, the loss of either gene or protein diminishing the activity of the complex. The remarkable manner in which the *trr* mutant alleles phenocopied the *Ptip* mutant alleles—disrupting Tin-lineage symmetric cardiac progenitor cell divisions and earlier symmetric Svp-lineage superprogenitor cell divisions while having little or no effect on Svp-lineage asymmetric progenitor cell divisions—suggested strongly that Ptip and Trr also functioned in this H3K4 HMT COMPASS-like complex to regulate symmetric cell division during embryonic cardiogenesis. This hypothesis was confirmed by the results of our genetic interaction assays comparing phenotypes of single and double heterozygotes of the *Ptip* and *trr* mutant alleles. Synergistic genetic interactions were detected between *Ptip* and *trr* for both Tin-lineage symmetric and Svp-lineage earlier symmetric cell divisions, but not for Svp-lineage asymmetric cell divisions, indicating that Ptip and Trr function together as a complex to regulate symmetric but not asymmetric cardiac cell divisions. Note that this deduced role of the Ptip-Trr-COMPASS-like complex in regulating symmetric but not asymmetric cardiac cell divisions is consistent with our previous discovery that individual homozygous mutations of *Ptip* and *trr* significantly affect symmetric but not asymmetric cell divisions.

Intriguingly, despite the homozygous mutations of *trr* and *Ptip* phenocopying one another, *trr/+* heterozygous hemisegments exhibited significantly more Tin-lineage symmetric cell division defects than the *Ptip/+* heterozygous hemisegments in our genetic interaction assays. These data indicate a cardiac requirement for proper *trr* gene dosage. The necessity of proper *trr* dosage likely reflects an evolutionarily conserved requirement for proper gene dosage of the mammalian *trr* ortholog, *KMT2D*, in human cardiac development since *KMT2D* haploinsufficiency is the cause of the developmental abnormalities involved in Kabuki syndrome, which include congenital heart defects [27,28,29,48].

Finally, even though the two other *Drosophila* COMPASS-like HMT-encoding genes *Set1* and *trx* also regulate cardiac cell divisions [19], additional genetic interaction assays revealed no synergistic genetic interactions between *Ptip* and either of these two genes. These results suggest that while all three *Drosophila* COMPASS-like complexes regulate cardiac cell division, the Ptip-Trr-COMPASS-like complex mediates this process through a mechanism that is distinct from that used by the Set1– or Trx–COMPASS-like complexes.

Our results extend previous findings defining critical functions for *Ptip*, *trr*, the Ptip-Trr-COMPASS-like complex, and the other COMPASS-like complexes in the regulation of cardiac gene expression via H3K4 methylation in post-embryonic heart development and adult heart function [1,5,6,19]. Together, these previous studies and our investigation of embryonic heart development confirm an important regulatory function for these genes in cardiac progenitor cell division. However, our investigation into the embryonic heart was not able to detect or establish a correlation between Ptip-Trr-COMPASS-like complex-mediated cardiac cell divisions and global cardiac H3K4me1 or H3K4me2 methylation: *Ptip* or *trr* homozygous mutations that produced symmetric cardiac cell division defects failed to show any effects on global H3K4me1 or H3K4me2 methylation. These results potentially implicate the Trr–COMPASS-like complex in processes independent of the global chromatin-modifying functions of the complex since H3K4 methylation was not significantly affected. Interestingly, *trr* and the mammalian orthologs *KMT2C/D* have been characterized as the key H3K4me1 HMTs necessary for maintaining active and poised enhancer regions of target genes [10,62,63]. Additionally, phenotypic and molecular studies of *Ptip* and *trr* truncation mutants in *Drosophila* have demonstrated that the transcriptional regulatory function and HMT activity are separable, suggesting distinct in vivo chromatin and transcriptional regulatory functions and the domains responsible for them [36,64]. However, further investigation at the molecular level, i.e., at a higher genomic resolution than was utilized in this study to evaluate chromatin changes at cardiac target gene loci, is necessary before one can conclusively assert that the Ptip–trr–COMPASS-like complex-regulated cardiac cell divisions are independent of the H3K4 methylation mediated by the complex.

In conclusion, our results identify a critical role for the Ptip-Trr-COMPASS-like complex in regulating the symmetric cardiac cell divisions that ensure proper cellular hemisegment patterning within the *Drosophila* embryonic heart tube, indicate that this function is distinct from the previously discovered cardiac cell division processes governed by the other two *Drosophila* COMPASS-like complexes, and suggest further that this process may be independent of the H3K4 methylation function also mediated by the Ptip-Trr-COMPASS-like complex.

## 4. Materials and Methods

### 4.1. Drosophila Strains and Genetics

The following mutant alleles and transgenes were utilized in these experiments were identified using FlyBase [65] and obtained from the Bloomington Drosophila Stock Center (Bloomington, IN, USA) when available [66]: *Ptip^MI03338^* [FlyBase ID: FBal0265465] [50], *Ptip^c450^* [FlyBase ID: FBal0345117, gift of Nan Liu and Rolf Bodmer] [51]; *trr^B^* [FlyBase ID: FBal0323346] [60]; *trr^C2375X^* [FlyBase ID: FBal0323346] [60]; *trx^E2^* [FlyBase ID:FBal0017174] [67]; *svp-lacZ* [*svp^3^*; FlyBase ID: FBal0016610] [57]; *Set1^G12^* [FlyBase ID: FBal0265880] [13]. The *Ptip* and *Set1* alleles were maintained over the *TM3, ftz-lacZ* balancer and *trr* alleles were maintained over the *FM7c, ftz-lacZ* X-chromosome balancer before crossing. Recombinants of *Ptip* mutants were generated to include the *svp-lacZ* enhancer trap on chromosome 3R. The embryos were genotyped by the absence of anti-β-galactosidase immunostaining in the *ftz-lacZ* pattern of the balancers, and the absence of Sex lethal (Sxl) for *trr* hemizygous male mutants and its presence for both female double heterozygotes of *Ptip* and *trr* and single heterozygotes of *trr*. Complementation tests were used to validate *Ptip^MI03338^* and *Ptip^c450^* mutant strains. Each *Ptip* strain failed to complement viability when crossed with the *Df(3L)ED4515* [FlyBase ID: FBab0035763] and *Df(3L)ED4536* [FlyBase ID: FBab0035784] deficiency strains. Additionally, the crosses between the two *Ptip* strains failed to complement each other.

### 4.2. Immunohistochemistry, Microscopy, and Cell Counting

Embryo fixation and fluorescent immunohistochemistry were performed as described previously examining protein expression [43,68]. However, for Svp protein immunostaining, heat fixation with embryo wash buffer was utilized. Briefly, embryos were boiled in embryo wash buffer (140 mM NaCl, 0.03% Triton^TM^ X-100, VWR Chemicals, Solon, OH, USA) for 10 s and immediately cooled with 4 °C embryo wash buffer before devitellinization in a 50:50 mixture of heptane and methanol, followed by storage in methanol before staining. The following primary antibodies were used: rabbit anti-Mef2 (1:1000, Clone ID: Mef2 from Developmental Studies Hybridoma Bank (DSHB, Iowa City, IA, USA) and gift from J. Jacobs), guinea pig anti-H15 (NMR1, 1:1000, gift from J. B. Skeath) [69], mouse anti-β-galactosidase (1:500, Catalog no. Z3783 [RRID:AB_430878] from Promega, Madison, WI, USA), chicken anti-β-galactosidase (1:500, Catalog no. ab9361 [RRID:AB_307210] from Abcam, Cambridge, UK), rabbit anti-H3K4me1 (1:1000, Catalog no. ab8895 [RRID:AB_306847] from Abcam), rabbit anti-H3K4me2 (1:1000, Catalog no. ab7766 [RRID:AB_2560996] from Abcam), mouse anti-Svp (1:5, Clone ID: 5B11 from the DSHB), and mouse anti-Sxl (1:20, Clone ID: M18 from the DSHB). DSHB antibodies were obtained from the Developmental Studies Hybridoma Bank, created by the National Institute of Child Health and Human Development (NICHD) of the National Institutes of Health (NIH) and maintained at The University of Iowa, Department of Biology, Iowa City, IA 52242. Fluorescent microscopy was performed on a Zeiss AxioImager with Apotome (Ziess, Oberkochen, Germany). Z-stacks of entire stage 16 embryonic hearts were scanned with a 20X or 40X objective and 0.60 µm and 0.31 µm steps, respectively, and used to quantify cells.

### 4.3. Statistical Methods

A comparison of cell division errors between genotypes using Fisher’s exact tests is not appropriate as the hemisegments for a given embryo are correlated in their likelihood of having such errors [40]. Comparison of cell division error rates between genotypes was thus performed using regression models with the response variable being the proportion of hemisegment errors for each embryo. Due to violation of regression assumptions, e.g., non-normality and heteroscedasticity, permutation (randomization) tests were used to obtain reliable *p*-values [70].

For comparing rates between two genotypes, for example, *trr^B^* and wild type, the following general linear model was used:

(1) $$Y_{j}=\beta_{0}+\beta_{1}\;I_{j}+\varepsilon_{j},$$

where $Y_{j}$ is the proportion of hemisegment errors for embryo j and the indicator variable $I_{j}$ is 1 if embryo *j* has phenotype *trr^B^* and 0 otherwise. To obtain a permutation *p*-value for testing $H_{0}:\;=\;0$, the estimate of $\beta_{1}$ for the actual data is compared with the estimates obtained when the genotypes of the embryos are permuted, i.e., the phenotype labels are randomly shuffled among the embryos in the sample. The permutation test *p*-value is then p=(n+1)/(N+1) where n is the number of permutation estimates for $\beta_{1}$ which exceed the estimate for the actual data and N is the number of permutations [71]. In order to obtain highly reproducible *p*-values, $N=10^{6}$ permutations were used for all permutation tests. All permutation tests were performed using R, version 4.2.2 (R Core Team, 2022).

For determining if cell division error rates are non-additively related to two gene mutations, for example, to detect synergistic interaction between *Ptip* and *trr*, a general linear model allowing for interaction was used:

(2) $$Y_{j}=\beta_{1}I_{p,j}+\beta_{2}I_{q,j}+\beta_{3}I_{p,j}I_{q,j}+\varepsilon_{j},$$

where $I_{p,j}$ is 1 if the *j*th embryo is heterozygous for the *Ptip^MI03338^* mutation and 0 otherwise, and $I_{q,j}=1$ only if it is heterozygous for the *trr^C2375X^* mutation. Since synergism is present only if $\beta_{3}\neq 0$, to detect it $H_{0}:\beta_{3}=0$ was tested using permutation. Since this is a multiple regression model, a somewhat more sophisticated permutation procedure, the Smith procedure (orthogonalization) was employed [72].

## Data Availability

The original contributions presented in the study are included in the article and Supplementary Material.

## Supplementary Materials

The following supporting information can be downloaded at: https://www.mdpi.com/article/10.3390/ijms26167954/s1.
