# Supplementary material for: Ptip and the Trr-COMPASS-like Complex Regulate Cardiac Progenitor Cell Division in the Drosophila Embryonic Heart Tube
Source: Int J Mol Sci. 2025 Aug 18;26(16):7954. doi: 10.3390/ijms26167954 (PMC12386242; doi:10.3390/ijms26167954)
Supplement: Supplementary file 1 [file ijms-26-07954-s001.zip › Figure S1.pdf]

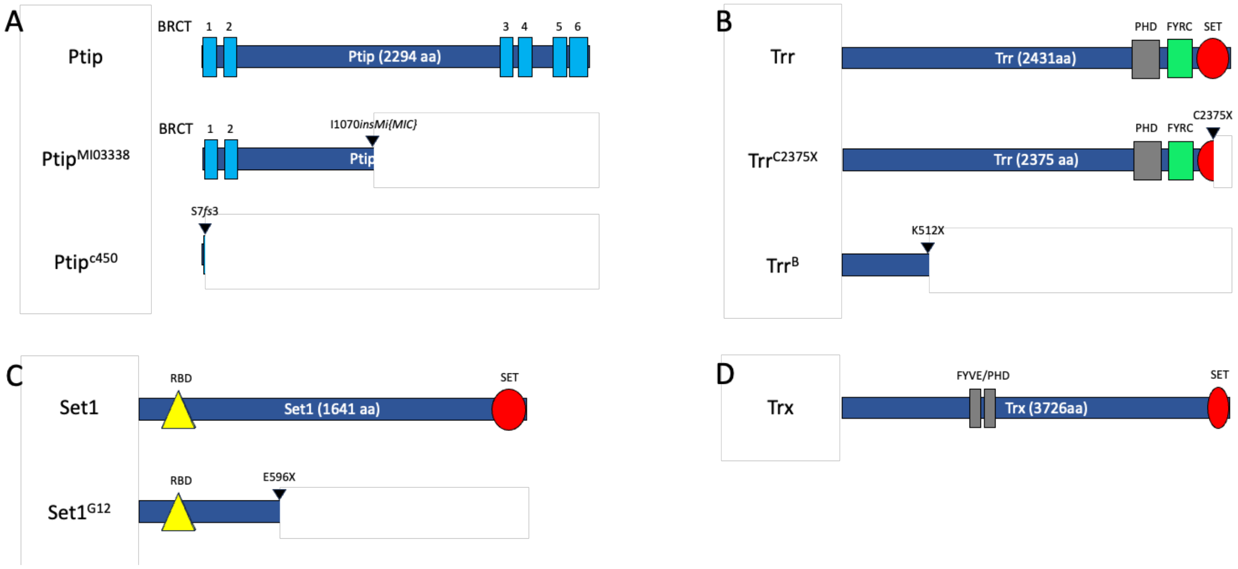

Figure S1. Predicted protein structural effects of mutant strains utilized within this study. The major domains identified by SUPERFAMILY are identified in each protein **(A)** The Ptip protein is a large protein consisting of two N-terminal and four C-terminal BRCT domains. The $Ptip^{MI03338}$ allele is predicted to generate a truncated protein due to the insertion of the *Mi{MIC}* transposon, while the $Ptip^{c450}$ *CRISPR/Cas9*-mediated allele is predicted to create a frameshift mutation abrogating a majority of the protein. **(B)** The Trr protein is a large protein consisting of a C-terminal PHD, FYRC, and a SET domain. The trr^{C2375} allele is predicted to generate a missense mutation that truncated protein within the c-SET sub-domain and POST-SET domains inactivating H3K4 methylation activity. The trr^{B} allele is also predicted to truncate the protein removing nearly 80% of the protein sequence. **(C)** Set1 significantly diverges in structure from both Trr and Trx proteins consisting of a N-terminal RNA-binding and C-terminal SET domain. The Set^{G12} allele is predicted truncate the protein removing more than half of the sequence. **(D)** Trx is a large protein consisting of FYVE/PHD domains and a C-terminal Set domain. The trx^{E2} allele is not illustrated since the nature of the lesion is unknown, however, thorough complementation tests with other *trx* hypomorphic, amorphic, and deficiency strains have provided significant evidence that this amorphic allele lack *trx* function compared to other *trx* alleles.
